# Therapeutic Time Migration in Hepatocellular Carcinoma: Rethinking the Timing of Systemic Therapy Across the Disease Continuum

**DOI:** 10.3390/cancers18183039

**Published:** 2026-09-19

**Authors:** Fausto Meriggi, Ester Oneda, Sara Cherri, Alberto Zaniboni

**Affiliations:** Oncology Department, Istituto Ospedaliero Fondazione Poliambulanza, Via Leonida Bissolati 57, 25124 Brescia, Italy

**Keywords:** hepatocellular carcinoma, systemic therapy, immunotherapy, TACE, TARE, thermal ablation, neoadjuvant therapy, therapeutic time migration, therapeutic hierarchy, temporal precision oncology

## Abstract

The management of hepatocellular carcinoma has traditionally followed a stage-dependent sequence in which surgery or thermal ablation is prioritized for early disease, transarterial approaches are used for intermediate-stage tumors, and systemic treatment is introduced after progression to advanced disease or loss of suitability for locoregional therapy. This paradigm was developed when systemic treatments had limited antitumor activity. Modern immune checkpoint inhibitors and immune–antiangiogenic combinations have substantially changed these premises. Randomized trials now demonstrate clinical benefit when systemic treatment is integrated with transarterial chemoembolization before conventional locoregional failure, while perioperative studies suggest that systemic treatment can also be moved into selected high-risk resectable disease. Conversely, updated adjuvant data indicate that simply administering systemic therapy at an earlier stage does not necessarily improve outcome after complete tumor eradication. We propose the concept of therapeutic time migration, whereby systemic treatment is introduced at an earlier biological point in selected patients, while hepatic reserve is preserved, occult systemic risk is becoming clinically relevant, and effective local treatment remains feasible. We explicitly distinguish this framework from the established concept of treatment stage migration and therapeutic hierarchy. This framework emphasizes treatment sequence rather than treatment escalation and suggests that timing itself may function as a dynamic therapeutic biomarker. Prospective studies should determine whether changing the order of systemic and locoregional therapy can modify immune activation, preserve liver function, increase conversion to definitive local treatment, and ultimately improve survival.

## Simple Summary

Hepatocellular carcinoma is traditionally treated according to tumor stage: surgery or ablation for early disease, liver-directed treatments for intermediate disease, and systemic therapy for advanced cancer. This sequence was developed when systemic treatments had limited efficacy. Modern immunotherapy-based combinations can produce substantial and sometimes durable tumor control, raising the question of whether systemic treatment should be introduced earlier in selected patients. This review proposes the concept of therapeutic time migration, in which treatment decisions consider not only tumor stage but also when systemic therapy is most likely to influence disease biology. Earlier integration with surgery, ablation, transarterial chemoembolization, or radioembolization may be advantageous when the risk of microscopic dissemination is high and liver function remains preserved. The objective is not to treat every patient earlier, but to identify the biological moment at which delaying systemic therapy may reduce future therapeutic opportunities.

## 1. Introduction

The most consequential question emerging in the contemporary management of hepatocellular carcinoma (HCC) may no longer be simply which systemic therapy should be used, but when it should be introduced. For decades, treatment has been organized according to a predominantly stage-dependent sequence. Patients with early tumors are considered for resection, transplantation, or thermal ablation; those with intermediate-stage disease are commonly treated with transarterial approaches; and systemic treatment has historically been reserved for vascular invasion, extrahepatic dissemination, or disease no longer amenable to effective locoregional control [1,2,3,4]. This therapeutic architecture was rational when systemic treatment produced limited tumor regression and relatively modest survival benefits. In such a setting, postponing systemic therapy until local options were exhausted represented a reasonable clinical strategy.

The systemic treatment landscape has since changed profoundly. Sorafenib first demonstrated that systemic therapy could prolong survival in advanced HCC, while lenvatinib subsequently provided an alternative first-line tyrosine kinase inhibitor [5,6]. The development of immune checkpoint inhibitors has produced a more fundamental change. Atezolizumab plus bevacizumab established the clinical value of combined immune and angiogenic inhibition, the STRIDE regimen demonstrated sustained survival beyond conventional median-based endpoints, and nivolumab plus ipilimumab has further confirmed that prolonged immune-mediated disease control can occur in a clinically relevant subgroup [7,8,9,10]. Other immune–antiangiogenic combinations have reinforced this therapeutic principle [11]. Systemic therapy is therefore no longer merely a late intervention intended to slow progression; in selected patients, it can produce deep and durable tumor control.

These advances have begun to challenge the traditional boundaries between local and systemic therapy. Randomized studies combining modern systemic treatment with transarterial chemoembolization (TACE) demonstrate that systemic therapy can improve disease control when initiated before conventional TACE failure [12,13]. At the other end of the disease spectrum, neoadjuvant and perioperative studies have shown that systemic therapy can be administered before definitive surgery and can induce substantial pathological tumor regression [14,15,16,17]. However, moving treatment earlier has not produced uniformly positive results. The initial recurrence-free survival advantage of adjuvant atezolizumab plus bevacizumab after curative-intent therapy was not maintained with longer follow-up [18,19]. Thus, chronological earliness and biological earliness may not be equivalent.

HCC also differs from many solid malignancies because the therapeutic window is determined by the simultaneous evolution of two diseases: the cancer and the underlying liver disorder. Tumor progression increases clonal complexity and the probability of dissemination, whereas repeated treatment, portal hypertension, and chronic liver disease can progressively erode hepatic reserve. Waiting therefore consumes both an oncological clock and a hepatic clock. We propose the term therapeutic time migration to describe the deliberate movement of systemic therapy toward an earlier biological phase in selected patients. Unlike conventional stage migration, this concept does not simply move treatments from one Barcelona Clinic Liver Cancer (BCLC) category into another. Instead, it considers timing and sequence as potentially modifiable determinants of therapeutic benefit.

This concept should be positioned in continuity with, rather than in opposition to, the established notions of treatment stage migration and therapeutic hierarchy in HCC. Vitale and colleagues described treatment stage migration as movement toward a therapeutic option associated with a neighboring BCLC stage when the stage-linked option is unsuitable, and subsequently developed a therapeutic hierarchy in which treatment choice is guided by expected benefit across a multiparametric profile rather than by anatomy alone [20,21]. Therapeutic time migration does not claim to replace these frameworks. Its proposed added value is narrower and longitudinal: after the preferred treatment has been identified through a therapeutic-hierarchy approach, it asks whether the biological value of that treatment may change according to when it is deployed relative to tumor evolution, deterioration of hepatic reserve, and local tumor eradication. In this sense, therapeutic hierarchy primarily addresses treatment selection, whereas therapeutic time migration is a hypothesis about treatment sequence and the preservation of therapeutic opportunity. Considerable overlap is expected, and the present framework should therefore be regarded as an extension of personalized therapeutic hierarchy into the temporal domain rather than as an entirely separate paradigm.

This article is a narrative, non-systematic review intended to generate and refine a clinical hypothesis rather than to provide a quantitative estimate of treatment effect. Relevant literature was identified through structured searches of PubMed/MEDLINE and Scopus (publications from 2008 to July 2026), using combinations of the terms “hepatocellular carcinoma”, “systemic therapy”, “immune checkpoint inhibitor”, “transarterial chemoembolization”, “radioembolization”, “neoadjuvant”, “adjuvant”, “perioperative”, “timing”, “tumor microenvironment”, and “treatment resistance”. Randomized phase II/III studies were prioritized for clinical claims; prospective single-arm studies were used primarily for feasibility and translational observations; mechanistic and pathology studies were used to support biological plausibility. Reference lists of major reviews and guidelines were hand-searched. Because no pre-registered protocol, formal risk-of-bias assessment, or GRADE evaluation was performed, selection bias and interpretive subjectivity remain unavoidable. To reduce overinterpretation, throughout the review we distinguish randomized clinical evidence, non-randomized clinical evidence, translational/mechanistic evidence, and hypothesis-generating inference, and Table 1 explicitly summarizes major study limitations.

## 2. Why Timing May Matter in HCC

The concept of therapeutic time migration should not be reduced to the intuitive assumption that treating cancer earlier is necessarily better. Its rationale in HCC derives from several specific biological and clinical features.

First, radiologically localized disease is not always biologically localized disease. Recurrence after apparently curative resection or ablation remains frequent, particularly in patients with adverse tumor characteristics. Microvascular invasion, satellite nodules, microscopic intrahepatic dissemination, and early metastatic clones may be present before they become radiologically detectable. Tumor size, multiplicity, vascular invasion, AFP level and kinetics, and pathological features all provide indirect information about this risk [22].

Consequently, local treatment and systemic treatment may target different dimensions of the same disease. Surgery, ablation, TACE, and TARE can provide highly effective control of identifiable tumor deposits, whereas systemic therapy potentially acts on malignant populations outside the local treatment field. The relevant distinction may therefore be less between anatomically “localized” and “advanced” disease than between disease that remains biologically local and disease that has acquired a systemic-risk phenotype.

Tumor evolution provides a second rationale. HCC progression can be accompanied by increasing intratumoral heterogeneity, hypoxia, angiogenesis, immune exhaustion, and selection of treatment-resistant clones. Although there is no definitive clinical evidence that checkpoint inhibition is intrinsically more effective at lower tumor burden in HCC, neoadjuvant studies provide important evidence that substantial immune remodeling can occur within a short period while the primary tumor remains intact [14,15,16,23].

A third consideration is unique in its clinical importance to liver cancer: systemic therapy may become less feasible over time even when the anticancer drug itself remains effective. The ALBI grade demonstrated that clinically meaningful heterogeneity of hepatic reserve exists even among patients conventionally classified as Child–Pugh A [24]. Repeated TACE can lead to progressive deterioration of hepatic function in a proportion of patients [25]. This means that a patient who is an excellent systemic-treatment candidate today may no longer be eligible for the same treatment after repeated ineffective locoregional interventions.

The disease trajectory therefore includes a systemic-treatment eligibility window. The clinical objective should not necessarily be to postpone systemic therapy for as long as technically possible, but to maximize lifetime tumor control while preserving the hepatic reserve required to exploit subsequent treatment options.

This consideration is particularly important in intermediate-stage disease. Historical definitions of TACE refractoriness were designed to identify the point at which continued embolization was unlikely to remain beneficial [26,27]. Modern treatment raises a different question: rather than asking when TACE has failed sufficiently to justify systemic therapy, should clinicians identify patients in whom the probability of repeated TACE failure is sufficiently high to justify systemic treatment before liver function deteriorates?

## 3. Moving Systemic Therapy Upstream: TACE as the First Proof of Principle

The strongest randomized evidence supporting earlier systemic integration currently comes from embolization-eligible HCC.

The historical approach to intermediate-stage disease was largely sequential:

- TACE → repeat TACE → further locoregional treatment → systemic therapy after refractoriness or stage progression.

This model implicitly assumes that systemic treatment retains the same therapeutic value regardless of when it is introduced. It also assumes that repeated locoregional therapy does not compromise subsequent systemic-treatment eligibility. Neither assumption is necessarily correct.

The idea of combining TACE and systemic therapy predates immunotherapy. The TACTICS trial showed that pre-treatment and continuation of sorafenib around TACE could prolong a TACE-specific progression endpoint compared with TACE alone, although the study did not establish a definitive overall-survival advantage [28]. The biological rationale for combination treatment became stronger with the development of immune–antiangiogenic therapy.

TACE induces ischemic tumor injury and necrosis but also produces hypoxia and substantial remodeling of the tumor microenvironment. Early histopathological observations reported increased VEGF expression in residual viable HCC after embolization [29], and a later pathology study described higher PD-1/PD-L1 expression in TACE-treated tumors [30]. These findings are biologically compatible with combining embolization with VEGF-pathway blockade and immune checkpoint inhibition, but their evidentiary strength should not be overstated: the VEGF report was a relatively small immunohistochemical study, and the PD-1/PD-L1 observation likewise derives from non-randomized tissue analyses. More recent reviews emphasize that TACE-related hypoxia, angiogenic signaling, myeloid-cell recruitment, immune suppression, and extracellular-matrix remodeling interact in a complex and context-dependent manner and may contribute both to therapeutic synergy and to resistance [31]. Accordingly, these translational data provide mechanistic plausibility rather than proof that a specific temporal sequence is clinically superior.

The phase III EMERALD-1 trial provided the first major randomized validation of this concept. Patients with unresectable HCC suitable for embolization were assigned to TACE with durvalumab plus bevacizumab, durvalumab without bevacizumab, or placebo. Median progression-free survival was 15.0 months with durvalumab plus bevacizumab compared with 8.2 months with TACE control, corresponding to a hazard ratio of 0.77 [12]. Importantly, durvalumab plus TACE without bevacizumab did not significantly improve the primary endpoint. This observation suggests that the interaction is more complex than simply combining checkpoint inhibition with tumor-antigen release; VEGF inhibition may be an important component of the biological partnership.

LEAP-012 independently reinforced this principle. Pembrolizumab plus lenvatinib combined with TACE improved median progression-free survival to 14.6 months compared with 10.0 months with TACE alone, with a hazard ratio of 0.66 [13]. However, the clinical cost of earlier systemic intensification was clear: severe treatment-related toxicity was substantially more frequent in the combination arm. The study therefore illustrates both the potential and the central limitation of therapeutic time migration. Earlier treatment is meaningful only when the predicted reduction in progression risk outweighs additional toxicity and the possibility of treating patients who could have achieved durable control with locoregional therapy alone.

Taken together, these studies redefine the conceptual position of systemic therapy. It is no longer introduced only because TACE has stopped working. Instead, it can be introduced while TACE remains capable of providing effective cytoreduction.

The relevant paradigm therefore changes from TACE refractoriness to TACE opportunity.

**Table 1:** Key clinical studies supporting or challenging earlier systemic treatment in HCC.

Clinical Setting	Study (Refs)	Sample Size	Phase/Design	Treatment Strategy	Main Result	Relevance to Therapeutic Timing	Key Limitations
Advanced/unresectable HCC	IMbrave150 [7]	*N* = 501	Phase III RCT	Atezolizumab + bevacizumab vs. sorafenib	Median OS 19.2 vs. 13.4 months in updated analysis	Established high activity of immune–antiangiogenic therapy	Advanced-disease trial; does not test timing or locoregional integration.
Unresectable HCC	HIMALAYA [8,9]	*N* = 1171	Phase III RCT	Durvalumab + Tremelimumab vs. sorafenib	Persistent OS advantage; 5-year OS 19.6% vs. 9.4%	Demonstrated durable immune-mediated disease control	Advanced-disease trial; does not test timing or locoregional integration.
Unresectable HCC	CheckMate 9DW [10]	*N* = 668	Phase III RCT	Nivolumab + ipilimumab vs. lenvatinib/sorafenib	Median OS 23.7 vs. 20.6 months	Reinforced potential for long-term systemic control	Advanced-disease trial; does not test timing or locoregional integration.
Unresectable HCC	CARES-310 [11]	*N* = 543	Phase III RCT	Camrelizumab + rivoceranib vs. sorafenib	Median OS 23.8 vs. 15.2 months	Further established immune–angiogenic synergy	Advanced-disease trial; does not test timing or locoregional integration.
Embolization-eligible HCC	TACTICS [28]	*N* = 156	Phase II RCT	TACE + sorafenib vs. TACE	Prolonged TACE-specific PFS/TTUP	Early proof that pre-TACE systemic treatment may modify the locoregional course	Phase II; TACE-specific progression endpoint; no definitive OS advantage.
Embolization-eligible HCC	EMERALD-1 [12]	*N* = 616	Phase III RCT	TACE + durvalumab + bevacizumab vs. TACE	Median PFS 15.0 vs. 8.2 months; HR 0.77	Phase III evidence for systemic integration before TACE failure	Tests treatment addition, not early-versus-late delivery; mature OS remains essential.
Embolization-eligible HCC	LEAP-012 [13]	*N* = 480	Phase III RCT	TACE + pembrolizumab + lenvatinib vs. TACE	Median PFS 14.6 vs. 10.0 months; HR 0.66	Independently validates early systemic–TACE integration	Tests treatment addition, not timing alone; increased severe toxicity.
Resectable high-risk HCC	CARES-009 [17]	*N* = 294	Phase II/III RCT	Perioperative camrelizumab + rivoceranib vs. surgery	Median EFS 42.1 vs. 19.4 months; HR 0.59	Randomized evidence for treatment beginning before surgery	Perioperative strategy vs surgery, not neoadjuvant vs identical adjuvant regimen; HBV-predominant Chinese cohort; OS immature.
High-risk HCC after resection/ablation	IMbrave050 [18,19]	*N* = 668	Phase III RCT	Adjuvant atezolizumab + bevacizumab vs. surveillance	Initial RFS benefit not maintained at later analysis	Shows that chronological earliness alone does not ensure sustained benefit	Adjuvant-only design; initial RFS benefit not sustained; no direct comparison with perioperative timing.
Liver-confined poor-prognosis HCC	HCRN GI15-225 [32]	Small (pilot)	Phase II, single-arm	Pembrolizumab initiated around Y90 radioembolization	Feasible; median OS 27.3 months in a small single-arm study	Supports investigation of sequence-specific immune–TARE combinations	Small single-arm pilot; no comparator; cannot isolate a sequence effect.

Cross-trial comparisons are descriptive only because populations, endpoints, disease stages, etiologies, and treatment strategies differ substantially. The limitations column distinguishes evidence for a specific regimen from evidence that timing itself is an independent causal variable.

## 4. Beyond TACE: TARE and Thermal Ablation as Partners for Systemic Therapy

The principle of systemic–locoregional integration extends beyond TACE.

Transarterial radioembolization (TARE) with yttrium-90 has evolved substantially with improvements in patient selection and dosimetry. Earlier systematic evidence already suggested clinically meaningful activity in intermediate and advanced HCC, while also highlighting marked heterogeneity in post-treatment liver decompensation and the predominantly non-randomized nature of the available evidence [33]. More recent studies have refined this experience: the LEGACY study demonstrated high and durable response rates with selective radioembolization in patients with solitary unresectable HCC [34], whereas DOSISPHERE-01 showed that personalized dosimetry can markedly increase objective response compared with standard dosimetry in locally advanced disease [35]. Longer follow-up has reinforced the potential clinical consequences of optimized radiation delivery [36]. These data support TARE as an important liver-directed modality, but they do not by themselves establish that adding or advancing systemic therapy improves outcome.

These developments make TARE particularly attractive as a partner for immunotherapy. Radiation can alter antigen presentation, induce tumor-cell death, release tumor-associated antigens, and remodel the immune microenvironment. The local effect of radiation could therefore provide a biological stimulus that systemic checkpoint inhibition might amplify.

Prospective clinical experience remains limited but informative. In CA 209-678, Y90 radioembolization followed by nivolumab produced objective responses in a meaningful subset of patients [37]. NASIR-HCC similarly demonstrated the feasibility of radioembolization followed by nivolumab and reported tumor responses that subsequently allowed surgery in selected patients [38].

The HCRN GI15-225 study is particularly relevant to the timing hypothesis because pembrolizumab was deliberately initiated before Y90 radioembolization [32]. Although the study was small, single-arm, and unable to establish superiority over either modality alone, its design demonstrates that immune therapy can be initiated while viable tumor remains present and before radiation-mediated antigen release occurs.

This distinction deserves prospective evaluation. A checkpoint inhibitor given before TARE encounters an intact tumor–immune interface, whereas the same drug started after radioembolization is delivered into a partially or extensively irradiated environment. Whether this sequence modifies systemic immune activation is unknown, but the question is biologically testable.

Thermal ablation provides an analogous experimental setting. Radiofrequency and microwave ablation can eradicate selected small tumors and should remain a standalone treatment for patients with favorable-risk disease. Routine immunotherapy around ablation is not currently justified.

However, thermal destruction releases tumor antigens and danger-associated molecular signals and creates a transient inflammatory environment. A proof-of-concept study suggested that ablation can alter subsequent responsiveness during PD-1 blockade [39]. This raises the possibility that selected high-risk tumors might benefit from a peri-ablative rather than purely adjuvant strategy.

The distinction is important. Conventional adjuvant therapy is initiated only after the macroscopic antigenic source has disappeared. Peri-ablative treatment could begin before destruction of the lesion, potentially allowing systemic immune priming to occur in the presence of the entire tumor-antigen repertoire.

At present, this hypothesis should remain clearly investigational. Its importance lies not in supporting routine peri-ablative immunotherapy today, but in identifying thermal ablation as an ideal model in which to study whether the order of local tumor destruction and immune activation influences subsequent disease control.

## 5. Resectable HCC: Neoadjuvant Therapy and the Adjuvant Paradox

Resectable HCC may provide the clearest biological setting in which to interrogate treatment timing.

Surgery can eliminate all radiologically identifiable disease, yet recurrence remains frequent in patients with adverse biological characteristics. Historically, preoperative systemic therapy was rarely considered because the probability of meaningful response was low and delaying potentially curative surgery was difficult to justify.

Modern immunotherapy has changed this balance.

Kaseb and colleagues demonstrated the feasibility of perioperative nivolumab with or without ipilimumab in resectable HCC and documented major pathological responses in a subset of tumors [14]. Marron et al. subsequently showed that even limited preoperative exposure to cemiplimab could induce extensive tumor necrosis [15].

Neoadjuvant cabozantinib plus nivolumab provided a particularly informative translational model. Twelve of 15 patients selected with locally advanced disease underwent margin-negative resection after treatment, with major pathological responses observed in several tumors [16]. Responding tumors demonstrated substantial immunological remodeling, supporting the concept that systemic therapy can modify the tumor–immune ecosystem before surgery.

More recently, CARES-009 moved the perioperative strategy into randomized testing. The study enrolled 294 patients with resectable HCC at intermediate or high risk of recurrence and compared surgery alone with two preoperative cycles of camrelizumab plus rivoceranib followed by surgery and postoperative continuation of the combination. Median event-free survival was 42.1 months in the perioperative group and 19.4 months with surgery alone, corresponding to a hazard ratio of 0.59 [17].

The result provides important proof of principle but should not be generalized uncritically. The trial population was predominantly HBV-associated and almost entirely Chinese; overall-survival data remain immature, and clinically significant toxicity occurred. Two deaths occurred during the neoadjuvant period [17]. Consequently, perioperative systemic therapy cannot yet be interpreted as a universal strategy for resectable HCC.

Its biological implications become particularly interesting when considered alongside adjuvant therapy.

IMbrave050 initially demonstrated an improvement in recurrence-free survival with one year of atezolizumab plus bevacizumab after curative-intent resection or ablation in patients considered at high risk of recurrence [18]. With longer follow-up, however, the difference attenuated. In the updated analysis, the recurrence-free survival hazard ratio was 0.90 and the original benefit was no longer sustained; overall survival remained immature [19].

This contrast raises what may be termed an “adjuvant paradox,” but it should be interpreted as a hypothesis-generating observation rather than a causal inference about timing. Adjuvant treatment is chronologically early, yet it begins after removal or destruction of macroscopic tumor, whereas neoadjuvant and perioperative treatment begins while an intact tumor and its antigenic repertoire remain present. Translational studies make it plausible that these settings differ immunologically, but CARES-009 and IMbrave050 cannot establish that the presence of the intact tumor, or the neoadjuvant component itself, caused the difference in clinical outcomes. The trials used different regimens, enrolled different populations, applied different recurrence-risk definitions, and differed substantially in etiology and design [17,18,19].

For immune checkpoint inhibition, the presence of an intact tumor may influence antigen presentation and tumor–immune interactions. Neoadjuvant HCC studies have documented rapid immune remodeling, and tertiary lymphoid structures (TLS) have been associated with pathological response and relapse-free outcomes after checkpoint blockade [23]. However, these associations are correlative. It remains uncertain whether TLS formation is a mediator of response, a consequence of effective immune activation, or a marker of a pre-existing immune-permissive phenotype; reproducibility across HBV-, HCV-, alcohol-, and MASLD (Metabolic Dysfunction-Associated Steatotic Liver Disease) -associated HCC is also not established. The hypothesis that pre-eradication immune priming is biologically advantageous is therefore testable and clinically interesting, but not proven.

The hypothesis is therefore that systemic therapy administered before tumor eradication may not be biologically equivalent to the same systemic therapy administered afterward.

This interpretation must remain cautious. CARES-009 did not compare a perioperative strategy with an identical adjuvant-only regimen, and cross-trial comparison with IMbrave050 cannot isolate the contribution of the neoadjuvant component. Drug combinations, populations, recurrence-risk definitions, and etiologies differ considerably.

Nevertheless, the contrast is sufficiently compelling to justify a new generation of trials in which timing itself becomes a randomized variable.

## 6. From Sequential Treatment to Response-Adapted Multimodal Care

Earlier systemic treatment has another potential advantage: it can reveal tumor biology before major local interventions are undertaken.

Under the traditional sequence, surgery, repeated embolization, or other locoregional treatments may be performed before systemic sensitivity is known. A systemic-first strategy reverses this order. Response becomes an in vivo biological test.

A patient who develops rapid extrahepatic progression despite effective systemic therapy has declared aggressive disease biology. In retrospect, extensive surgery or repeated liver-directed interventions might have provided little durable benefit.

Conversely, a patient who achieves major tumor regression, favorable AFP kinetics, disappearance of vascular invasion, and prolonged absence of new lesions may represent a biologically selected candidate for definitive local consolidation.

This concept expands the traditional meaning of conversion therapy. Conversion should not refer exclusively to anatomical reduction sufficient to make surgery technically feasible. It should also include biological conversion, in which a period of systemic therapy demonstrates disease behavior compatible with durable local control.

Potential consolidative approaches after systemic induction may include resection, ablation, selective TACE, personalized TARE, or stereotactic radiotherapy according to tumor distribution and liver function.

The therapeutic trajectory may therefore become:

- systemic induction → early biological and radiological reassessment → selective definitive local treatment → response-adapted systemic continuation or discontinuation.

Such an approach also generates an apparent paradox: introducing systemic therapy earlier could ultimately allow less cumulative systemic therapy.

In advanced HCC, treatment is often continued until progression or unacceptable toxicity. If systemic therapy instead produces major disease control followed by complete local consolidation, selected patients could theoretically enter a treatment-free interval rather than remain on indefinite therapy. This possibility is not yet validated and should be tested prospectively, but it reinforces the distinction between therapeutic time migration and treatment intensification.

The goal is not to maximize exposure. It is to use each modality when its biological value is highest. Table 2 summarizes this proposed framework across the HCC continuum.

## 7. Discussion: From Stage Migration to Therapeutic Time Migration

The emerging HCC literature suggests that the historical separation between systemic and locoregional treatment is becoming increasingly artificial. The major conceptual change is not simply the movement of immunotherapy from BCLC C toward BCLC B or selected BCLC A populations. More fundamentally, the biological context in which systemic therapy is initiated may influence its therapeutic value.

This temporal perspective overlaps substantially with treatment stage migration and therapeutic hierarchy [20,21]. We therefore view therapeutic time migration as a complementary refinement rather than a competing construct. Therapeutic hierarchy helps determine which treatment is expected to provide the greatest net benefit for a given multiparametric profile; therapeutic time migration asks whether that net benefit may change if the same modality is delivered before versus after a clinically meaningful transition such as repeated TACE, loss of hepatic reserve, or local tumor eradication. The distinction is conceptual and remains to be prospectively validated.

Stage migration is primarily anatomical. A treatment originally developed for one stage is applied to a neighboring stage. Therapeutic hierarchy ranks available treatments by expected efficacy for a given multiparametric profile at a single point in time. Therapeutic time migration is longitudinal: it considers whether the same therapy may have a different effect depending on tumor burden, hepatic reserve, previous treatment exposure, immune context, and the presence or absence of viable macroscopic tumor.

The current evidence does not prove that timing is an independent determinant of treatment efficacy. EMERALD-1 and LEAP-012 tested treatment addition to TACE rather than earlier versus later delivery of an identical systemic regimen [12,13], and CARES-009 tested a perioperative strategy against surgery alone rather than isolating the preoperative component [17]. These trials therefore support earlier systemic integration as a clinical strategy in selected settings, but they cannot distinguish a true timing effect from the effects of treatment intensification, drug interaction, or patient selection. The evidence for therapeutic time migration should consequently be separated into three levels: (i) established clinical evidence that particular multimodal regimens improve defined outcomes; (ii) mechanistic or translational evidence that makes sequence biologically plausible; and (iii) the unproven hypothesis that changing treatment order while holding treatment exposure constant will improve clinically meaningful outcomes.

First, the most convincing randomized evidence for moving systemic therapy earlier has emerged when macroscopic disease remains present. EMERALD-1 and LEAP-012 integrate systemic treatment while TACE is still being performed [12,13]. CARES-009 begins systemic treatment before surgery [17].

Second, updated adjuvant-only evidence has been less convincing. The loss of the early recurrence-free survival separation in IMbrave050 suggests that simply exposing patients to checkpoint inhibition soon after curative treatment is insufficient to guarantee sustained disease control [18,19].

Third, the HCC treatment window can close because of declining liver function rather than tumor progression alone. This makes treatment sequencing fundamentally different from that of cancers arising in otherwise healthy organs. A technically feasible locoregional intervention may still carry a future cost if it reduces the hepatic reserve required for subsequent systemic therapy [24,25].

The heterogeneity of HCC makes universal early treatment inappropriate. A solitary small HCC with favorable biology and high probability of cure through ablation or surgery does not justify systemic treatment merely because an active regimen exists. Therapeutic time migration therefore requires selection, not expansion for its own sake.

The clinically relevant threshold might ultimately be defined as the point at which the expected risk of microscopic dissemination, multifocal failure, or rapid recurrence exceeds the expected toxicity and opportunity cost of systemic therapy.

Traditional risk variables, including tumor size and number, vascular invasion, AFP, histological differentiation, recurrence interval, and radiological progression pattern, can contribute to this assessment [22]. However, the future of temporal selection will probably require more dynamic biomarkers.

Circulating tumor DNA (ctDNA) is particularly attractive. Postoperative ctDNA characteristics have been associated with recurrence after HCC resection [41], and tumor-informed assays have demonstrated feasibility for detecting molecular residual disease and anticipating clinical relapse [42]. These approaches remain investigational, but they illustrate how biological risk may ultimately be measured rather than inferred.

Radiomics may provide another layer. Models developed to predict microvascular invasion show promising performance, although methodological heterogeneity and limited external validation currently prevent routine clinical implementation [43].

Immune biomarkers may be equally informative. Neoadjuvant studies have shown that tertiary lymphoid structures and associated immune-cell organization correlate with pathological response and relapse-free outcomes after checkpoint inhibition [23]. Such markers could eventually help identify tumors in which immune priming before local eradication is particularly valuable.

These developments point toward a shift from static anatomical staging to dynamic biological staging.

A patient could therefore be characterized not simply as having early or intermediate HCC, but as having either:

- anatomically localized and biologically localized disease, or
- anatomically localized but biologically systemic-risk disease.

The second category is where therapeutic time migration is most likely to become clinically relevant.

However, several limitations must be acknowledged. A progression-free survival improvement does not necessarily imply an overall-survival benefit, particularly when effective therapies can be given after progression. Moving systemic therapy earlier may merely alter treatment order rather than increase lifetime survival. Mature survival follow-up is therefore essential.

As a narrative rather than systematic review, this work is also subject to methodological limitations inherent to non-systematic literature synthesis. Study selection was not governed by a pre-registered protocol, reflects the authors’ judgment regarding relevance and topical fit, and did not include a formal risk-of-bias or certainty-of-evidence assessment (e.g., using GRADE or a comparable framework) of the individual studies discussed. These limitations should be kept in mind when weighing the strength of the conclusions drawn, and they should be addressed through systematic and living-review approaches as the underlying evidence base matures.

Toxicity is equally important. The increase in severe adverse events observed with systemic–TACE combination therapy illustrates the danger of assuming that additional disease control necessarily improves the overall therapeutic index [13]. Perioperative systemic treatment similarly exposes potentially curable patients to treatment-related complications that would not occur with surgery alone [17].

Etiological heterogeneity is central to the external validity of any temporal-treatment framework. Much of the strongest perioperative evidence, particularly CARES-009, derives from Asian populations dominated by HBV-associated HCC [17]. By contrast, HCV-, alcohol-, and MASLD-related HCC account for a large and growing proportion of Western disease. These etiologies differ in background inflammation, fibrosis, metabolic comorbidity, immune-cell composition, and competing risks of liver- and cardiovascular-related mortality. The HCC microenvironment is itself heterogeneous, with regulatory T cells, myeloid-derived suppressor cells, tumor-associated macrophages, stromal elements, angiogenic signaling, metabolic programs, and potentially neuro-immune interactions contributing to immune escape and treatment resistance [31,44]. Recent work linking FTH1-driven ferritinophagy and tryptophan metabolism to HCC progression further illustrates how metabolic state can influence tumor biology, although these findings remain preclinical and are not yet treatment-selection biomarkers [45]. Thus, temporal strategies validated in HBV-predominant cohorts should not be assumed to have identical benefit–risk profiles in MASLD-, HCV-, or alcohol-associated HCC. Future trials should stratify prospectively by etiology and incorporate liver-related and cardiovascular competing events, rather than treating etiology as a descriptive baseline variable.

A practical framework must also protect patients from overtreatment. Earlier systemic therapy should not be triggered by stage alone and should not be considered when a patient has a high probability of durable control with resection, ablation, transplantation, or a limited course of locoregional therapy. Until prospectively validated thresholds exist, candidate selection should be framed as a multidimensional risk–benefit assessment rather than as a recommendation for routine early treatment. Features that may increase the rationale for investigation include macro- or microvascular invasion risk, multifocal or rapidly recurrent disease, high or rising AFP, large tumor burden, unfavorable radiomic features, early recurrence after prior curative therapy, incomplete or short-lived response to locoregional treatment, and a trajectory suggesting imminent loss of ALBI-defined hepatic reserve. Conversely, transplant eligibility, low-risk solitary disease, substantial autoimmune or bleeding risk, frailty, and major competing comorbidity should raise the threshold for systemic exposure. No acceptable false-positive rate for this strategy has yet been defined; prospective enrichment studies should therefore report both unnecessary systemic exposure and loss of subsequent therapeutic opportunities caused by delayed treatment.

Liver transplantation constitutes a particularly important exception. Immune checkpoint inhibition before transplantation may increase the risk of allograft rejection and should only be considered within multidisciplinary protocols involving transplant hepatology, surgery, oncology, and interventional radiology. An individual patient-data meta-analysis found that longer washout was associated with lower rejection risk; a median interval of approximately 94 days was associated with a predicted rejection probability of ≤20%, supporting a pragmatic target of about 3 months when oncologically feasible [40]. This threshold is based on retrospective and pooled observational evidence rather than randomized data and should not be interpreted as an absolute guarantee of safety. Therapeutic time migration must never compromise a more valuable curative pathway.

Two further dimensions deserve explicit consideration. First, the incremental cost of immune-based combinations is substantial, and moving them earlier could magnify drug acquisition, monitoring, toxicity-management, and opportunity costs across a larger population. Cost-effectiveness analyses in advanced HCC already show wide variation across regimens and healthcare systems. For example, in one Chinese-payer model, atezolizumab plus bevacizumab generated an estimated total cost of US $104,188 and 0.84 quality-adjusted life-years, with an ICER of approximately US $160,049 per QALY versus sorafenib; these values are highly setting- and price-dependent and should not be extrapolated directly to other health systems [46]. Equivalent analyses for TACE-integrated, neoadjuvant, and perioperative strategies are largely absent. Economic evaluation should therefore be embedded prospectively and should include the cost of avoided or repeated locoregional procedures, hospitalizations, management of immune-related adverse events, and downstream treatment sequencing. Second, earlier systemic exposure may affect quality of life even when conventional efficacy endpoints improve. Future trials should prospectively incorporate validated patient-reported outcome instruments such as the EORTC QLQ-C30 with an HCC-specific module where appropriate, FACT-Hep, EQ-5D, and time-to-deterioration analyses. Health-related quality of life has been preserved or improved with some immune–antiangiogenic regimens in advanced disease [47], but this cannot be extrapolated automatically to patients who might otherwise have required local therapy alone.

The concept should therefore be understood as therapeutic synchronization rather than therapeutic escalation.

The objective is not to use more treatments. It is to combine local and systemic modalities at the time when each is most likely to contribute to durable disease control while preserving subsequent therapeutic possibilities.

## 8. Timing as a Therapeutic Biomarker

Biomarkers are conventionally defined as measurable characteristics that provide prognostic information or predict sensitivity to therapy. In HCC, truly predictive molecular biomarkers remain relatively uncommon. Even in the modern systemic era, treatment choice is largely determined by clinical variables, liver function, disease distribution, contraindications, and previous treatment rather than by tumor genotype.

Timing may be better regarded, at present, as a candidate composite decision variable rather than a validated biomarker. It is not a molecular alteration and cannot be measured by a single assay. Operationalization would require a prespecified score or algorithm integrating tumor risk, hepatic reserve, disease trajectory, and future treatment eligibility, followed by prospective validation for discrimination, calibration, reproducibility, and, most importantly, predictive interaction with treatment sequence. Candidate components include AFP level and kinetics, ctDNA or molecular residual disease, radiomic predictors of microvascular invasion, serial ALBI grade, radiological growth rate, pathological response, immune gene signatures, T-cell clonality, spatial immune features, and TLS organization [23,41,42,43]. None currently defines a validated threshold for moving systemic therapy earlier.

Instead, it reflects a dynamic biological state generated by the interaction of tumor characteristics, liver function, host immunity, previous therapy, and future treatment eligibility.

The therapeutic substrate therefore changes over time even when the drug remains identical.

A checkpoint inhibitor given while macroscopic tumor is intact acts in the presence of a large and heterogeneous antigenic reservoir, an active tumor microenvironment, and organized tumor–immune interactions. The same drug administered after complete resection is delivered in a context where only microscopic disease may remain.

Similarly, systemic therapy initiated in a patient with preserved ALBI grade before repeated TACE is not biologically equivalent to the same systemic regimen administered months later after deterioration of hepatic reserve.

The treatment is unchanged, but the system in which it acts has changed.

This suggests that timing could be conceptualized as a composite dynamic therapeutic biomarker incorporating several domains.

Tumor biology could include burden, distribution, growth rate, vascular invasion, AFP kinetics, molecular profile, and radiomic predictors.

Hepatic reserve could include ALBI grade, portal hypertension, synthetic function, and, importantly, the direction of liver-function change over time rather than a single baseline value.

Disease trajectory could incorporate recurrence interval, response to prior locoregional treatment, development of new intrahepatic sites, and speed of progression.

Immune context could eventually include T-cell clonality, immune gene signatures, tertiary lymphoid structures, myeloid populations, and spatial characteristics of the tumor microenvironment.

Molecular residual disease could be incorporated through ctDNA or other liquid-biopsy approaches [41,42].

Finally, future treatment eligibility should itself be recognized as dynamic. A patient may simultaneously be eligible for surgery, embolization, immunotherapy, and several subsequent systemic regimens at one point in the disease course. A sequence of ineffective treatments may progressively eliminate these options.

Timing therefore captures not only what the disease is today, but what the disease and the patient may become if treatment is deferred.

The difference from a conventional predictive biomarker can be expressed through two questions.

A conventional biomarker asks:

*“Is this tumor likely to respond to this treatment?”*

A temporal biomarker asks:

*“Is this the biological moment at which this treatment has its greatest potential value?”*

This hypothesis is directly testable.

In resectable HCC, future trials could compare an identical systemic regimen delivered perioperatively with the same total treatment exposure delivered only postoperatively. Such a design would isolate the contribution of preoperative immune priming.

In ablatable HCC, the same systemic treatment could begin before versus after thermal ablation.

In TARE-based strategies, checkpoint inhibition could be initiated before or after radioembolization.

In TACE-eligible disease, studies could investigate whether systemic priming before the first embolization produces a different biological and clinical outcome from systemic treatment beginning only after TACE.

Importantly, these studies should integrate translational endpoints capable of demonstrating whether timing changes biology. Pathological response, ctDNA clearance, expansion of tumor-reactive lymphocyte populations, tertiary lymphoid structures, spatial immune remodeling, and dynamic liver-function measurements could complement conventional efficacy endpoints.

This leads to a broader concept of temporal precision oncology.

Conventional precision oncology seeks the right treatment for the right patient. In HCC, precision may require an additional dimension:

- the right treatment, for the right patient, combined with the right local intervention, at the right biological time.

Timing may therefore prove to be more than a scheduling decision. It may represent an integrated measure of therapeutic opportunity.

## 9. Research Priorities and Roadmap

Translating therapeutic time migration from a conceptual framework into clinical practice will require a coordinated research agenda. The following priorities summarize the actionable steps outlined throughout this review:

Before clinical implementation, any proposed temporal-selection algorithm should be prospectively locked and validated in independent cohorts. A clinically useful model would need to identify a group with sufficiently high risk of recurrence, multifocal failure, or loss of hepatic reserve that the absolute benefit of earlier systemic therapy exceeds treatment-related toxicity, financial burden, and the probability of unnecessary exposure. Randomized interaction testing is ultimately required to demonstrate that the selected group benefits specifically from an altered sequence rather than simply from receiving additional therapy.

- Randomize treatment order, not only treatment addition. Design trials in which an identical cumulative systemic exposure is delivered before versus after local tumor control across resectable, ablatable, TACE-eligible, and TARE-eligible settings.
- Incorporate dynamic biomarkers. Use ctDNA/molecular residual disease, radiomics, serial ALBI grade, and tertiary lymphoid structure assessment as stratification factors and translational endpoints, rather than relying solely on baseline anatomical staging.
- Prioritize overall survival (OS) and competing-risk-aware endpoints. OS should remain the definitive endpoint for practice-changing timing trials. Progression free survival (PFS), event-free survival (EFS), time to untreatable progression, loss of systemic-treatment eligibility, conversion to definitive local therapy, and treatment-free survival are informative complementary endpoints, but should not substitute for mature OS. Analyses should account for post-progression crossover, subsequent effective therapies, liver-related death, and non-cancer mortality, particularly cardiovascular events in metabolically driven HCC.
- Broaden etiological representation. Extend evaluation of therapeutic time migration beyond predominantly HBV-associated Asian cohorts to populations with metabolic dysfunction-associated steatotic liver disease, HCV, and alcohol-associated cirrhosis.
- Integrate economic and patient-reported outcomes prospectively, so that incremental clinical benefit from earlier systemic integration can be weighed against cost and quality-of-life impact [46,47].
- Safeguard curative pathways. Define clear criteria for excluding potential liver-transplant candidates from immune-checkpoint-based time-migration strategies, given the documented risk of post-transplant graft rejection [40].

## 10. Conclusions and Future Directions

The traditional therapeutic architecture of HCC placed systemic treatment near the end of the disease pathway. Localized disease was managed locally, and systemic therapy was generally introduced only when local control was no longer feasible.

That sequence reflected the efficacy of the treatments available when the model was developed.

It may no longer fully reflect the therapeutic potential of modern HCC care.

Immune-based systemic regimens have demonstrated substantial antitumor activity and durable survival in advanced disease [7,8,9,10,11]. Randomized trials also show that specific systemic combinations can improve disease control when integrated with TACE before conventional locoregional failure [12,13], and CARES-009 provides randomized evidence that a perioperative strategy can improve event-free survival in selected high-risk resectable disease [17]. These observations establish the clinical feasibility and potential value of earlier multimodal integration; they do not yet prove that timing itself is the causal determinant of benefit.

At the same time, updated adjuvant evidence demonstrates that chronological earliness does not guarantee sustained benefit [19]. IMbrave050 should not be interpreted as proving biological inferiority of adjuvant treatment relative to neoadjuvant treatment; rather, together with perioperative data, it generates a testable question about whether treatment sequence, intact-tumor immune priming, regimen choice, or patient selection modifies outcome.

The more important question may be:

- At what point in the biological evolution of HCC does delaying systemic treatment begin to reduce the probability of long-term disease control?

Answering this question will require prospective trials specifically designed around treatment order rather than simply treatment addition. Such studies should determine whether initiating systemic therapy before local tumor eradication modifies immune responses, whether earlier systemic integration prevents deterioration of hepatic reserve, and whether response-adapted local consolidation can increase durable treatment-free survival.

Several ongoing or recently completed trials are beginning to address this question directly. The Notable-HCC phase 1b trial (NCT05185531) tested neoadjuvant PD-1 blockade combined with stereotactic body radiotherapy before resection in early-stage resectable HCC, demonstrating feasibility and encouraging pathological responses without surgical delay [48]. The ongoing phase III EMERALD-2 trial (NCT03847428) is evaluating adjuvant durvalumab with or without bevacizumab after curative resection or ablation, providing a further test of whether chronological earliness alone translates into sustained recurrence-free benefit. Dedicated trials in which timing itself, rather than treatment addition, is the randomized variable remain comparatively rare and represent a priority for the field, as outlined in Section 9.

Future trials should also define endpoints appropriate for multimodal sequencing. OS remains indispensable and should be prioritized for definitive practice-changing conclusions, particularly because PFS can be affected by treatment order, post-progression crossover, and subsequent therapy. Complementary endpoints, including time to untreatable progression, deterioration of ALBI grade, loss of systemic-treatment eligibility, conversion to definitive local treatment, molecular residual disease, pathological response, and treatment-free survival, may clarify mechanism and patient benefit. Competing risks from liver failure and non-cancer mortality should be prespecified, especially in patients with cirrhosis and metabolic or cardiovascular comorbidity.

The objective of therapeutic time migration is not systemic therapy earlier for everyone. It is systemic therapy before the therapeutic window closes in patients whose biological risk makes waiting potentially detrimental.

If prospective studies demonstrate that treatment sequence independently influences immune activation, hepatic-function preservation, conversion to definitive therapy, and survival, timing itself may become a clinically actionable therapeutic biomarker.

**Figure 1:**
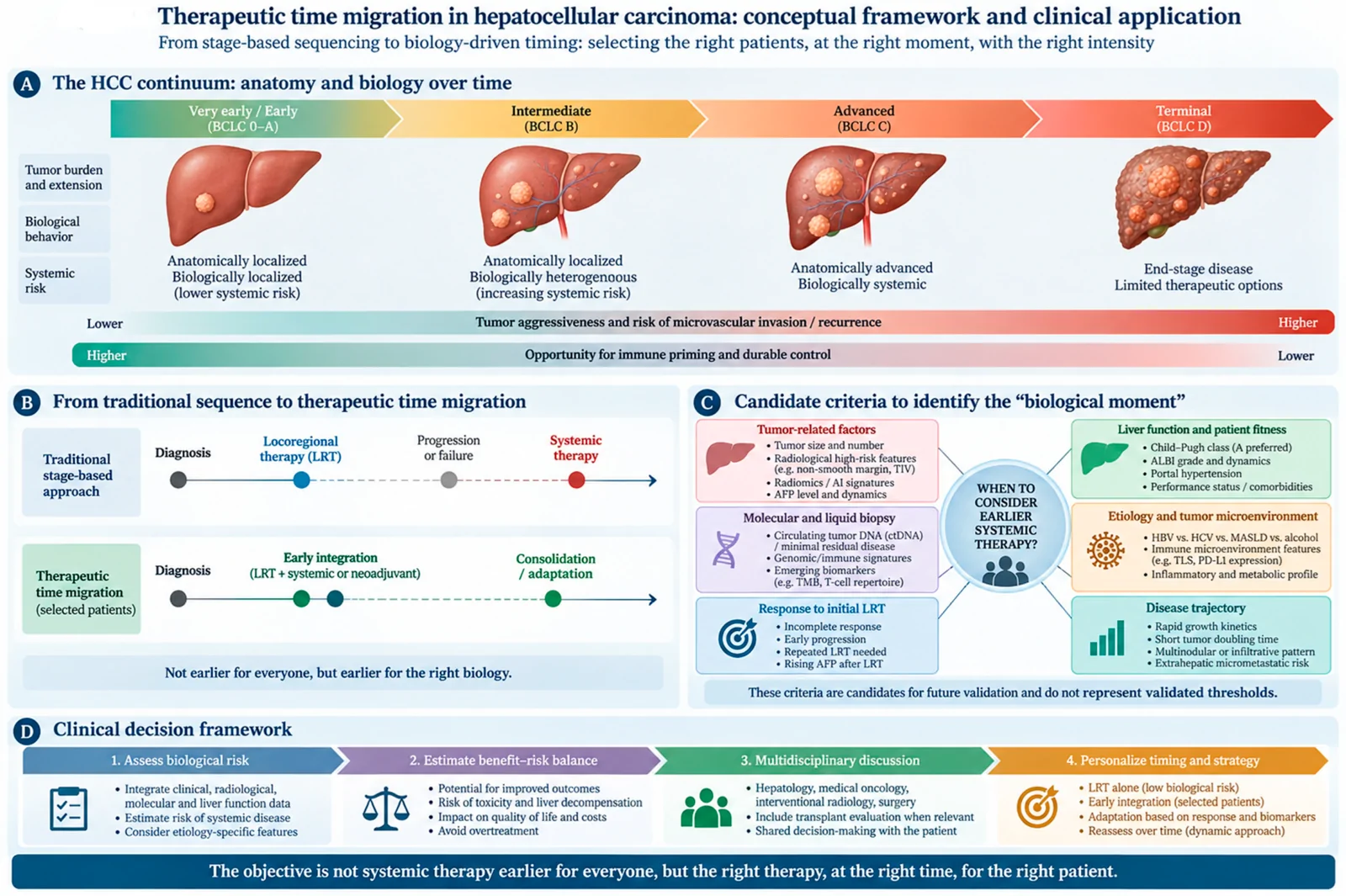
From Traditional Sequential Therapy to Therapeutic Time Migration in HCC. Conceptual model. Systemic treatment may have greater value when introduced while hepatic reserve is preserved, effective local therapy remains feasible, and the probability of systemic or multifocal failure has become clinically relevant. Candidate research settings include high-risk resectable HCC, selected TACE-eligible disease with high risk of multifocal failure or hepatic-reserve loss, TARE-based multimodal strategies, and response-adapted conversion after systemic therapy. The model does not imply routine systemic treatment for all patients with early-stage HCC.

The transition from stage migration and therapeutic hierarchy [20,21] to therapeutic time migration could then represent a new dimension of HCC management: the emergence of temporal precision oncology. Figure 1 summarizes this conceptual framework and its clinical application.

Central message: the clinically relevant transition may not be from local to systemic disease, but from biologically localized to biologically systemic-risk disease.

## Figures and Tables

**Table 2:** Proposed framework for therapeutic time migration across the HCC continuum.

Clinical Scenario	Conventional Approach	Temporally Integrated Strategy	Main Rationale	Current Evidence
Very early/low-risk HCC	Ablation or resection → surveillance	Local therapy alone	High probability of durable local control; avoid overtreatment	Established standard; no rationale for routine systemic therapy.
High-risk resectable HCC	Surgery → surveillance or trial-based postoperative treatment	Short systemic priming → surgery → risk/response-adapted postoperative treatment	Early occult-disease control; biological selection; pathological response assessment	Randomized perioperative signal; trial-based/selected use only. Candidate enrichment: vascular invasion risk, multifocality, AFP kinetics, radiomics, ctDNA/MRD, pathological response.
High-risk ablatable HCC	Ablation → surveillance	Systemic priming → ablation → selected consolidation	Potential antigen release and immune priming	Investigational; avoid routine systemic exposure when ablation alone is highly likely to cure.
TACE-eligible intermediate HCC	TACE → repeat TACE → systemic treatment after failure	Systemic treatment integrated with initial TACE	Simultaneous control of visible and occult disease; reduced reliance on repeated TACE	Phase III PFS-positive combinations; balance benefit against toxicity, liver reserve, and likelihood of durable TACE control.
High-burden or TACE-unsuitable liver-confined HCC	Variable repeated liver-directed therapy or systemic treatment	Systemic-first → response-adapted local consolidation	Protect hepatic reserve; reveal disease biology before repeated intervention	Biologically plausible but selection unresolved; prioritize preserved liver function and avoid futile repeated LRT.
TARE-eligible disease	TARE alone or sequential systemic treatment	Immunotherapy before or around TARE	Potential radiation–immune interaction	Early prospective evidence only; sequence effect unproven.
Initially unresectable disease with major systemic response	Continue systemic therapy	Selective surgery/ablation/TARE after durable response	Anatomical and biological conversion	Investigational; require durable systemic control and multidisciplinary confirmation of consolidative value.
Potential liver-transplant candidate	Bridging/downstaging LRT	Checkpoint inhibition only within highly selected multidisciplinary protocols	Potential post-transplant allograft rejection	Special caution: preserve transplant candidacy; ICI only in multidisciplinary protocols with adequate washout when feasible [40].

## Data Availability

No new data were created or analyzed in this study. Data sharing is not applicable to this article.
